# Hybrid Vision Perception for Low-Cost Autonomous Vehicle Navigation

**DOI:** 10.3390/s26206626

**Published:** 2026-10-19

**Authors:** Abeeb Akorede Bello, Sreedhar Madichetty, Bonginkosi Thango, Abdelkader El Kamel

**Affiliations:** 1 Department of Electrical and Computer Engineering, Mahindra University, Hyderabad 500043, Telangana, India; 2 Department of Electrical and Electronic Engineering Technology, Faculty of Engineering and Built Environment, University of Johannesburg, Johannesburg 2092, South Africa; 3 Centrale Lille Institute, University of Lille, 59000 Lille, France; abdelkader.elkamel@centralelille.fr

**Keywords:** autonomous vehicles, machine vision algorithm, ArUco markers, image-processing techniques, planning and localization

## Abstract

**Highlights:**

**What are the main findings?**

- The hybrid perception system combines ArUco localization, Canny edge detection, and YOLOv4 object detection to generate forward, turning, and stopping commands on a commercially available four-wheel electric vehicle platform fitted with the prototype perception and control system.
- Among the evaluated edge detectors, Canny achieved the highest performance, with a precision of 0.99 and a recall of 0.98, supporting its use within the navigation pipeline.

**What are the implications of the main findings?**

- A low-cost monocular vision architecture can provide useful localization, boundary detection, and obstacle awareness without requiring a large LiDAR-, RADAR-, or multi-camera sensor suite.
- The successful laboratory and controlled outdoor deployments demonstrate the feasibility of integrating lightweight classical vision and deep object detection for resource-constrained autonomous navigation platforms.

**Abstract:**

High-performance perception systems for autonomous vehicles (AVs) commonly use LiDAR, RADAR, and multiple cameras. These sensors provide useful information, but their cost and computational requirements can be difficult to justify for small prototypes and low-cost platforms. This work therefore uses a monocular camera and combines three vision methods: Canny edge detection for boundary information, ArUco markers for localization, and YOLOv4 for object detection. The outputs are used to generate navigation commands on a commercially available four-wheel electric vehicle platform equipped with the prototype control system. The experiments compare the individual image-processing methods and evaluate the complete system under different visual conditions. The results indicate that the combined approach can provide useful localization and obstacle detection without requiring a large sensor suite.

## 1. Introduction

The autonomous vehicle (AV) field has moved rapidly over the last few years, and it is already changing how transportation works day to day—creating safer roads, reducing congestion, and providing mobility options that were not really practical before [1]. Underneath all of it lies one hard requirement: the vehicle has to know where it is and what is around it because, without that, meaningful autonomy is simply unachievable [2,3,4]. At present, the dominant approach is a heavy one—LiDAR, RADAR, several cameras, and deep learning stitched on top to make sense of it all. It undoubtedly performs well, but the price tag and the computational demands it carries make it a non-starter for cost-sensitive work, such as research in university labs, small prototypes, or AV deployments aimed at low-income areas [5,6]. A low-cost vehicle still needs localization and obstacle information when GPS is unavailable, such as in tunnels, underpasses, and areas with limited satellite visibility. LiDAR-based localization and SLAM can address these conditions, but they add hardware and processing requirements. A camera-based alternative can reduce this complexity if the limitations of image-only sensing are considered during system design [7,8]. This paper presents a hybrid vision system for localization and navigation. ArUco markers are used for pose estimation, while Canny edge detection provides boundary information. Both methods have relatively low computational requirements. The marker information is used to determine the vehicle position with respect to the navigation markers, while the edge information is used in the decision logic. Canny edge detection can be affected by illumination changes, image noise, and motion blur. To provide additional information in such cases, a pretrained YOLOv4 detector is included in the system. YOLOv4 is used to identify objects such as pedestrians and vehicles. The detected objects are then considered together with the marker and edge information before a vehicle command is issued.

### 1.1. Motivation

The main motivation is to develop a perception system that can be implemented on a relatively low-cost platform. A complete LiDAR, RADAR, and multi-camera setup may not be practical for small research vehicles or teaching platforms because of the additional hardware and processing requirements [9,10]. A camera-based system using lightweight image processing can reduce these requirements while still providing the information needed for basic navigation and obstacle detection [10,11,12]. The proposed system combines ArUco localization and Canny edge detection with YOLOv4 object detection. ArUco and Canny provide the basic geometric information used by the navigation logic, while YOLOv4 supplies object-level information. The three outputs are processed on the onboard computer and used to generate commands for the vehicle.

### 1.2. Literature Review

Machine vision is widely used in autonomous vehicle perception for object detection, lane detection, and localization. Many systems combine cameras with LiDAR and RADAR to improve environmental awareness [13]. The use of several sensors, however, increases the requirements for calibration, data processing, and hardware. Several studies have examined low-cost computing platforms for vision applications. Kaymak and Ucar demonstrated the use of Raspberry Pi and Arduino platforms with SIFT, SURF, FAST, and ORB algorithms for detection and localization [14]. Hasan presented an intelligent decision-making system for traffic congestion management using adaptive routing and online data processing [3]. These studies show that lower-cost computing platforms can be considered for selected perception and decision-making tasks. Deep-learning methods have also been applied to online object detection. YOLO was developed for single-stage object detection with an emphasis on fast inference [15], and later work has considered its use in driving environments [16]. Edge detection and Hough-transform methods remain common for lane identification [17]. Other deep-learning approaches have been used for depth estimation, obstacle detection, and vehicle behavior modeling [18,19]. Learning-based control methods have also been investigated for collision avoidance and adaptive vehicle decisions [20]. These methods generally require more training data and computational resources than classical image-processing methods. Fiducial markers provide another option for camera-based localization. ArUco markers are widely used because their encoded IDs can be detected from image data and their corners can be used for pose estimation. ArUcoE introduced additional processing to improve marker detection and pose estimation [16]. ArUco-based visual localization has also been reported for aerial landing applications [21]. In vehicle applications, marker position and size can be used as navigation information [22]. Canny edge detection is also used for extracting road and lane boundaries, including under low-light conditions [23]. Its output can be combined with localization information to support navigation decisions. Previous work has also considered combinations of classical and learning-based detection for adverse weather conditions [22].

The connection between perception and vehicle control is also important. Fuzzy-logic and PD-based controllers have been used for trajectory and stability control in mobile robotic systems [24]. This work therefore considers the perception output together with the low-level motor-control stage. The reviewed studies indicate that both classical and deep-learning methods have useful roles in vehicle perception. Classical methods generally require less computation, while deep-learning methods provide object-level information. Based on these observations, this study combines ArUco localization, Canny edge detection, and YOLOv4 object detection. The approaches and their reported limitations are summarized in Table 1.

The practical contribution of the present work is not a new individual detector; it is the integration of marker-based localization, edge-based path information, and object-level obstacle detection into a single decision stage connected to the vehicle controller. This allows the system to use complementary information from methods with different computational requirements. Unlike marker-only navigation, the marker is not the sole decision source: boundary information and obstacle information can override or modify the vehicle command.

### 1.3. Key Contributions

- Hybrid Perception Framework: A perception system combining ArUco marker localization, Canny edge detection, and YOLOv4 object detection on a resource-constrained vehicle platform.
- Comparative Evaluation: Canny, Sobel, Prewitt, and Roberts edge detectors are compared using precision and recall, and the perception methods are evaluated as part of the vehicle decision process.
- Hardware Experimental Validation: The prototype perception and control system was integrated with a commercially available four-wheel electric vehicle platform using an onboard processor, microcontroller, and an I2C motor-driver interface and tested during indoor and controlled outdoor trials.

### 1.4. Organization

The remainder of the paper is organized as follows. Section 2 describes the proposed methodology. Section 3 presents the experimental results, and Section 4 gives the conclusions.

## 2. Methodology

### 2.1. Problem Statement

**Figure 1:**
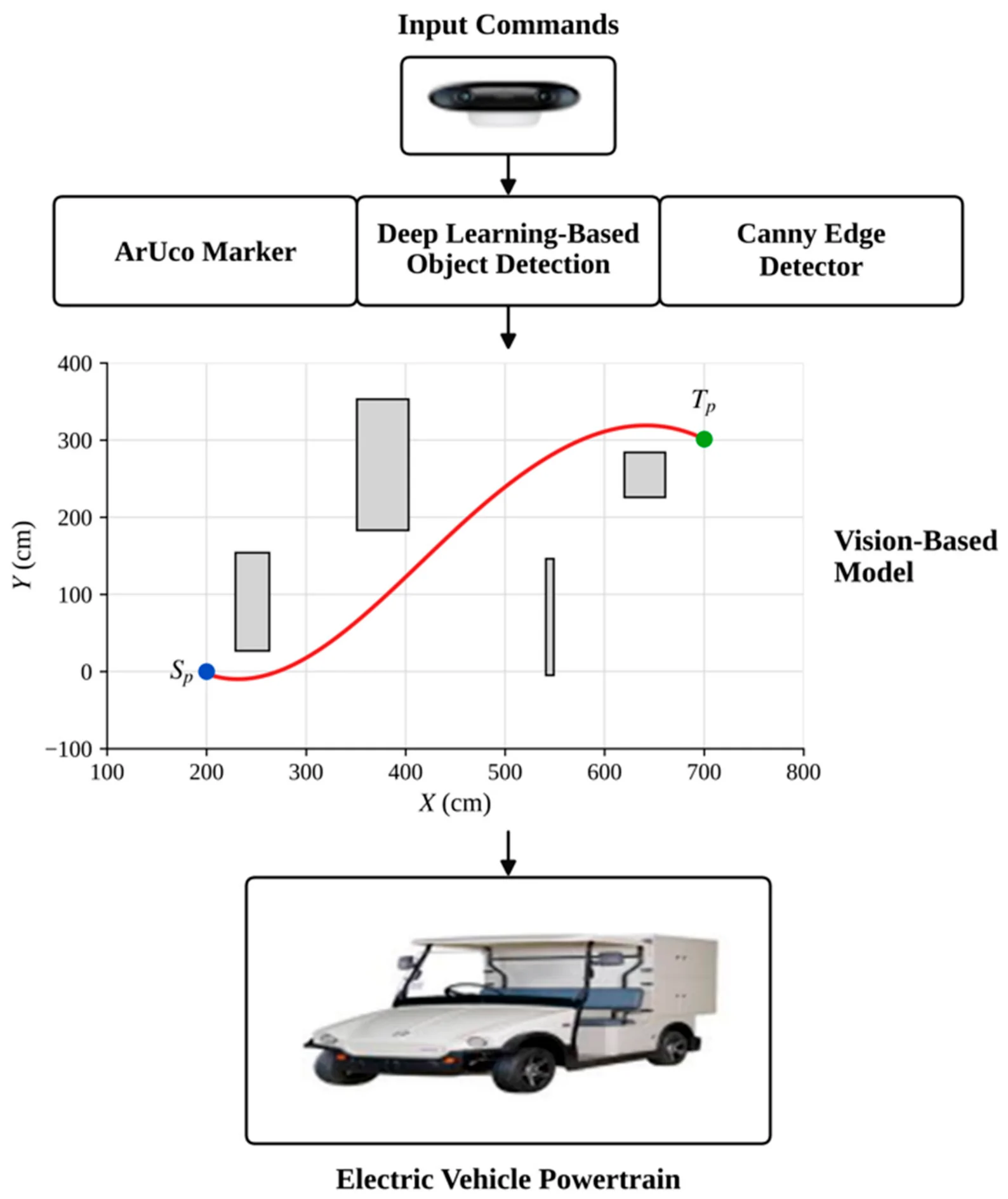
The machine vision algorithm for AV.

The objective is to implement a camera-based perception and localization system with limited computational requirements. The four-wheel electric vehicle used for the experiments was a commercially available market platform; the vision-based perception, decision, communication, and motor-control integration developed in this study constitute the research prototype. ArUco markers are used for localization, and Canny edge detection is used to obtain boundary information. YOLOv4 is added for object detection. The overall arrangement is shown in Figure 1.

#### 2.1.1. Marker-Based Pose Estimation

**Figure 2:**
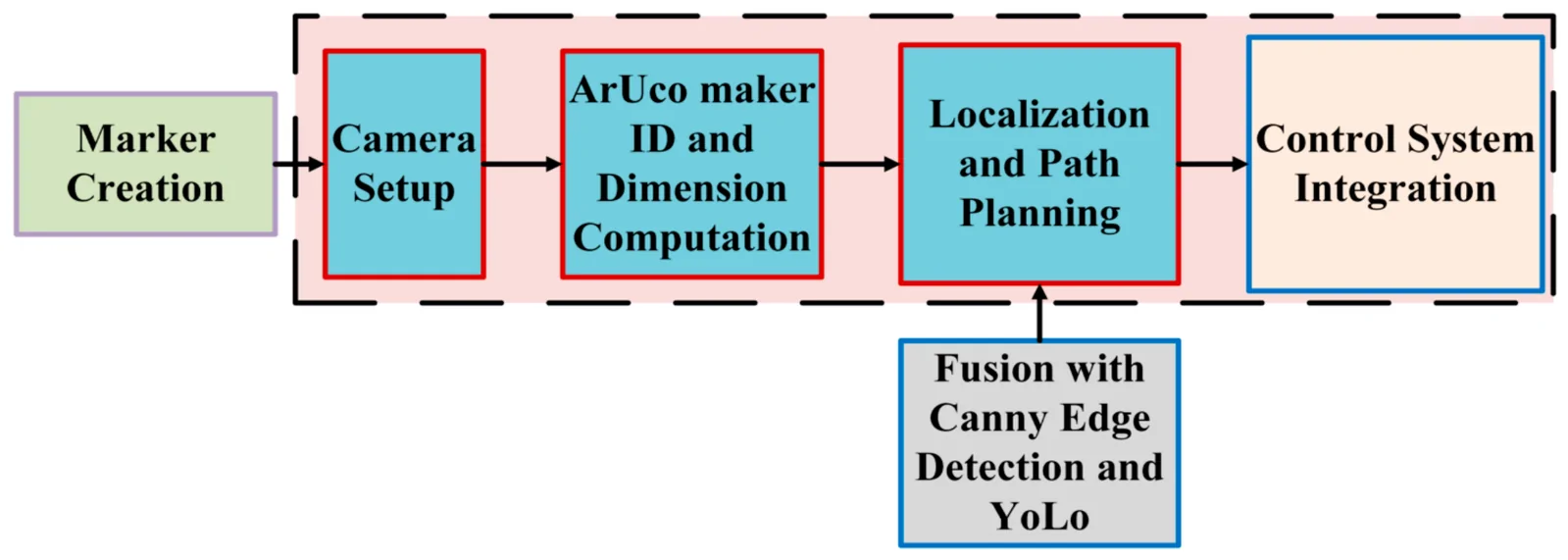
ArUco marker setup.

The ArUco setup is shown in Figure 2. Each marker contains a binary ID within a square pattern. The four marker corners, $P_{i}$, $\left(i=1,2,3,4\right)$, are detected in the image and are used to estimate the marker pose with respect to the camera using Equation (1):

(1) $$p_{i}=K\left(RP_{i}+t\right)$$

Here, $p_{i}$ represents the image point corresponding to the 3-D point $P_{i}$. The matrices R and t represent the marker rotation and translation, respectively, and K is the camera intrinsic matrix given in Equation (2):

(2) $$K=\left[\begin{array}{ccc} f_{x} & 0 & C_{x}\\ 0 & f_{y} & C_{y}\\ 0 & 0 & 1 \end{array}\right]$$

The parameters $f_{x}$ and $f_{y}$ are the focal lengths, while $C_{x}$ and $C_{y}$ define the principal point. The marker pose is obtained by solving the Perspective-n-Point (PnP) relation in Equation (3), where $\left(U_{i},V_{i}\right)$ are image coordinates and $\left(X_{i},Y_{i},Z_{i}\right)$ are the corresponding 3-D coordinates. The variable s is the projection scale factor:

(3) $$s\left[\begin{array}{c} U_{i}\\ V_{i}\\ 1 \end{array}\right]=K\left[R|t\right]\left[\begin{array}{c} X_{i}\\ Y_{i}\\ Z_{i}\\ 1 \end{array}\right]$$

The marker-to-camera distance is estimated using Equations (4)–(6). Here, f is the camera focal length and L is the physical marker size. The image dimensions $w_{image}$ and $h_{image}$ are obtained from the detected marker. A smaller image size corresponds to a larger estimated distance.

GPS was not used in the reported navigation loop. GPS can improve global position awareness and route-level navigation, but it does not replace the local visual information provided by ArUco and Canny. A future implementation can combine GPS for global localization with camera-based local positioning; reference-station corrections could also be used to obtain more accurate position information.

(4) $$w=max\left(x_{1},x_{2},x_{3},x_{4}\right)-min\left(x_{1},x_{2},x_{3},x_{4}\right)$$

(5) $$h=max\left(y_{1},y_{2},y_{3},y_{4}\right)-min\left(y_{1},y_{2},y_{3},y_{4}\right)$$

(6) $$D=\frac{f\cdot L}{\frac{w_{image}+h_{image}}{2}}$$

#### 2.1.2. Edge-Based Scene Understanding

**Figure 3:**
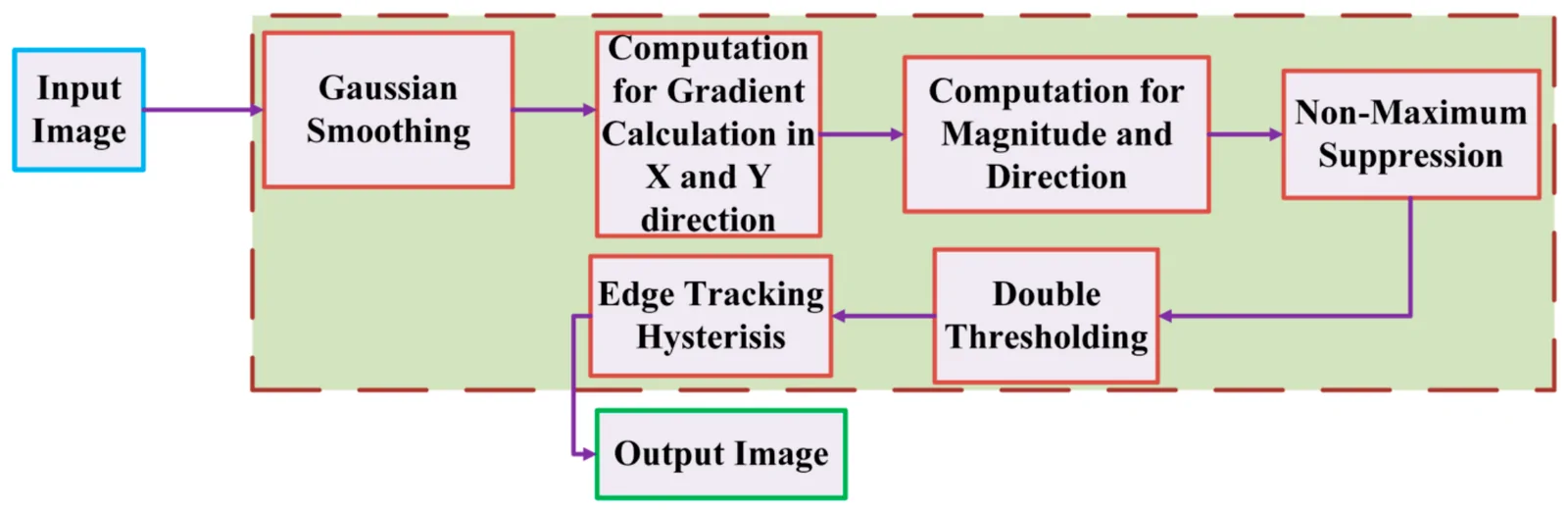
Canny edge detection algorithm.

Canny edge detection is processed in parallel with marker detection. The processing steps are shown in Figure 3.

First, the RGB image is converted to grayscale using Equation (7):

(7) $$Gray\left(x,y\right)=0.299\cdot R\left(x,y\right)+0.587\cdot G\left(x,y\right)+0.114\cdot B\left(x,y\right)$$

The image gradients in the horizontal and vertical directions are then calculated using Equations (8) and (9):

(8) $$G_{x}\left(x,y\right)=\frac{\partial I\left(x,y\right)}{\partial x}=I\left(x+1,y\right)-I\left(x-1,y\right)$$

(9) $$G_{y}\left(x,y\right)=\frac{\partial I\left(x,y\right)}{\partial y}=I\left(x,y+1\right)-I\left(x,y-1\right)$$

In these equations, $G_{x}\left(x,y\right)$ and $G_{y}\left(x,y\right)$ are the horizontal and vertical gradients, respectively, and $I\left(x,y\right)$ is the grayscale intensity. The gradient magnitude and direction are calculated using Equations (10) and (11) [25]:

(10) $$G\left(x,y\right)=\sqrt{G_{x}{\left(x,y\right)}^{2}+G_{y}{\left(x,y\right)}^{2}}$$

(11) $$\Theta \left(x,y\right)=arctan\left(\frac{G_{y}\left(x,y\right)}{G_{x}\left(x,y\right)}\right)$$

A low threshold of $T_{L}=50$ and a high threshold of $T_{H}=150$ were used for the Canny detector. These values were selected experimentally after several test runs. The 1:3 threshold ratio provided a suitable compromise between removing weak noise and retaining connected edges in the tested images. The edge map $E\left(x,y\right)$ is subsequently dilated to increase the width of the detected boundaries. The operations are represented by Equations (12) and (13):

(12) $$E\left(x,y\right)=\left\{\begin{array}{ll} 1, & G\left(x,y\right)>150\\ 0.5, & 50<G\left(x,y\right)\leq 150\\ 0, & G\left(x,y\right)\leq 50 \end{array}\right.$$

(13) $$E_{d}\left(x,y\right)={max}_{\left(i,j\right)\in K}E\left(x+i,y+j\right)$$

The number of edge pixels calculated from the dilated image is used by the navigation logic together with the marker information, as given in Equation (14):

(14) $$N_{edges}=\sum_{x=1}^{H}\sum_{y=1}^{W}1\;\left(E_{d}\left(x,y\right)=255\right)$$

#### 2.1.3. Process Pipeline

The image-processing routines were first developed and tested in MATLAB 2024b. ArUco detection was developed separately in Python 3.15.0 using an Anaconda Jupyter Notebook. The two parts were then combined and deployed on an NVIDIA Jetson Nano 4 GB embedded board, identified by the recorded hardware configuration of a 128-core Maxwell GPU, quad-core ARM Cortex-A57 CPU, and 4 GB of 64-bit LPDDR4 memory. The processor sends encoded commands to the microcontroller through I2C, and the microcontroller generates PWM signals for the motor-control stage. The available study records do not include a purchase price or bill-of-materials breakdown; therefore, the low-cost description is used only to describe the reduced sensor configuration, and no specific monetary savings are claimed.

The control chain consists of the NVIDIA Jetson Nano 4 GB, a microcontroller, and motor drivers. I2C is used between the processor and microcontroller, while PWM is used for motor control. A separate steering-angle measurement card is not part of the reported configuration. The archived experimental record does not contain the Jetson nvpmodel power-mode setting or timestamped stage-wise/end-to-end timing logs, so those quantities cannot be reconstructed reliably after the experiment.

### 2.2. Deep Learning-Based Perception

YOLOv4 was included to provide object information that is not available from marker and edge detection alone. This is particularly useful when the image contains pedestrians, vehicles, or other objects that need to be considered by the navigation logic. YOLOv4 processes the input image in a single forward pass. The network uses CSPDarknet53 for feature extraction and PANet for multi-scale feature fusion. The detector produces bounding-box coordinates $\left(x,y,w,h\right)$, an objectness score $P_{obj}$, and a class confidence $P_{class_{i}}$. The resulting confidence is calculated using Equation (15):

(15) $$P_{conf}=P_{obj}\cdot P_{class_{i}}$$

Non-Maximum Suppression (NMS) is then applied to remove overlapping bounding boxes using the IoU measure in Equation (16):

(16) $$IoU=\frac{Area\left(B_{pred}\cap B_{gt}\right)}{Area\left(B_{pred}\cup B_{gt}\right)}$$

The selected IoU threshold determines which overlapping detections are retained. The present implementation was evaluated for the reported forward, right-turn, and stop cases. A left-turn command was not experimentally evaluated. The decision framework can be extended by adding a turn-direction condition derived from marker/path geometry; this extension is identified as future work rather than a tested result.


(17) $$\begin{array}{c} \mathrm{Cmd}=\\ \mathrm{STOP},\;\mathrm{if}\;O=1\\ \mathrm{TURN}\;\mathrm{RIGHT},\;\mathrm{if}\;M=1\wedge E=1\wedge O=0\\ \mathrm{FORWARD},\;\mathrm{if}\;M=1\wedge E=0\wedge O=0\\ \mathrm{STOP},\;\mathrm{otherwise} \end{array}$$

#### 2.2.1. Performance Evaluation

The system was tested using daylight, low-light, and cluttered scenes from the custom road dataset. Image and video samples were processed using the Canny–ArUco methods and YOLOv4. For the edge comparison, the reported precision and recall values were calculated from the available test images; the original study did not preserve a separate manually annotated pixel-level ground-truth mask or a formal annotation protocol. These values are therefore treated as an evaluation of the available test set rather than a statistically generalizable benchmark. A larger manually annotated dataset with a predefined annotation protocol is required for a rigorous edge detection benchmark.

(18) $$Precision=\frac{TP}{TP+FP},\;Recall=\frac{TP}{TP+FN}$$

Here, TP, FP, and FN denote true positives, false positives, and false negatives. The experimental observations were then used to assess the behavior of the combined perception system.

#### 2.2.2. Marker Pose and Distance Estimation

Marker pose estimation is used during navigation to obtain the marker position relative to the camera. For the 3-D marker coordinates $\left(X_{i},Y_{i},Z_{i}\right)$ and image coordinates $\left(U_{i},V_{i}\right)$, the projection relation is given in Equation (19):

(19) $$\left[\begin{array}{c} U_{i}\\ V_{i}\\ 1 \end{array}\right]=K\left[R|t\right]\left[\begin{array}{c} X_{i}\\ Y_{i}\\ Z_{i}\\ 1 \end{array}\right]$$

Here, K is the intrinsic camera matrix, R the rotation matrix, and t the translation vector connecting world and image coordinates. The distance D is calculated from Equation (6) using the camera focal length, marker size, and detected image dimensions. This distance is used by the navigation process as the vehicle approaches the marker. The complete decision procedure is given in Algorithm 1.
**Algorithm 1:** Hybrid decision-making for autonomous vehicle navigation**Data:** Camera frames F, ArUco dictionary, edge threshold $T_{edges}$, YOLOv4 detector**Result:** Vehicle movement commandInitialize: camera, ArUco settings, YOLOv4 detector;while destination not reached do  Capture frame F; convert to grayscale G;  Apply Canny edge detection → edges E; dilate E;  Count edge pixels: $N_{edges}$;  Detect ArUco markers in G → IDs, dimensions;  Detect objects in F using YOLOv4 → class labels;  MarkerValid ← False; ObstacleDetected ← False;  foreach marker do    if $w_{marker}\geq 17$ and $h_{marker}\geq 19$ then      MarkerValid ← True;    end  end  foreach YOLO detection do    if class ∈ {person, car, bicycle, motorbike} then      ObstacleDetected ← True;    end  end  if ObstacleDetected then    Command ← STOP  else if MarkerValid && $N_{edges}>T_{edges}$ then    Command ← TURN RIGHT  else if MarkerValid && $N_{edges}\leq T_{edges}$ then    Command ← MOVE FORWARD  else    Command ← STOP  end  Display F, E, detections, and decision;  if user presses ‘q’ then    break;  endend

## 3. Results

**Figure 4:**
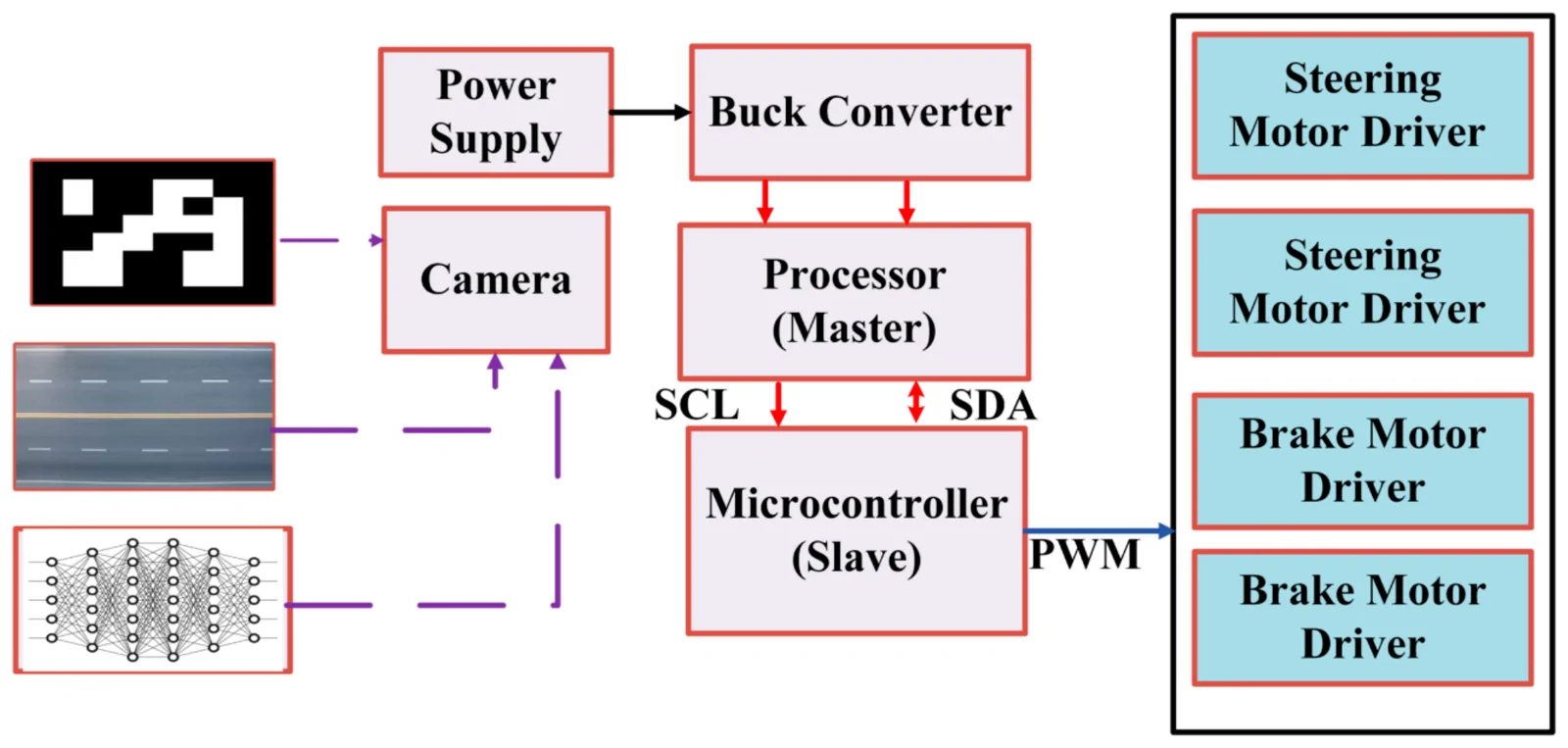
Block diagram of the system.

This section presents the experimental results obtained from the image-processing methods, the decision logic, and the embedded vehicle platform shown in Figure 4.

### 3.1. Edge Detection Performance

**Figure 5:**
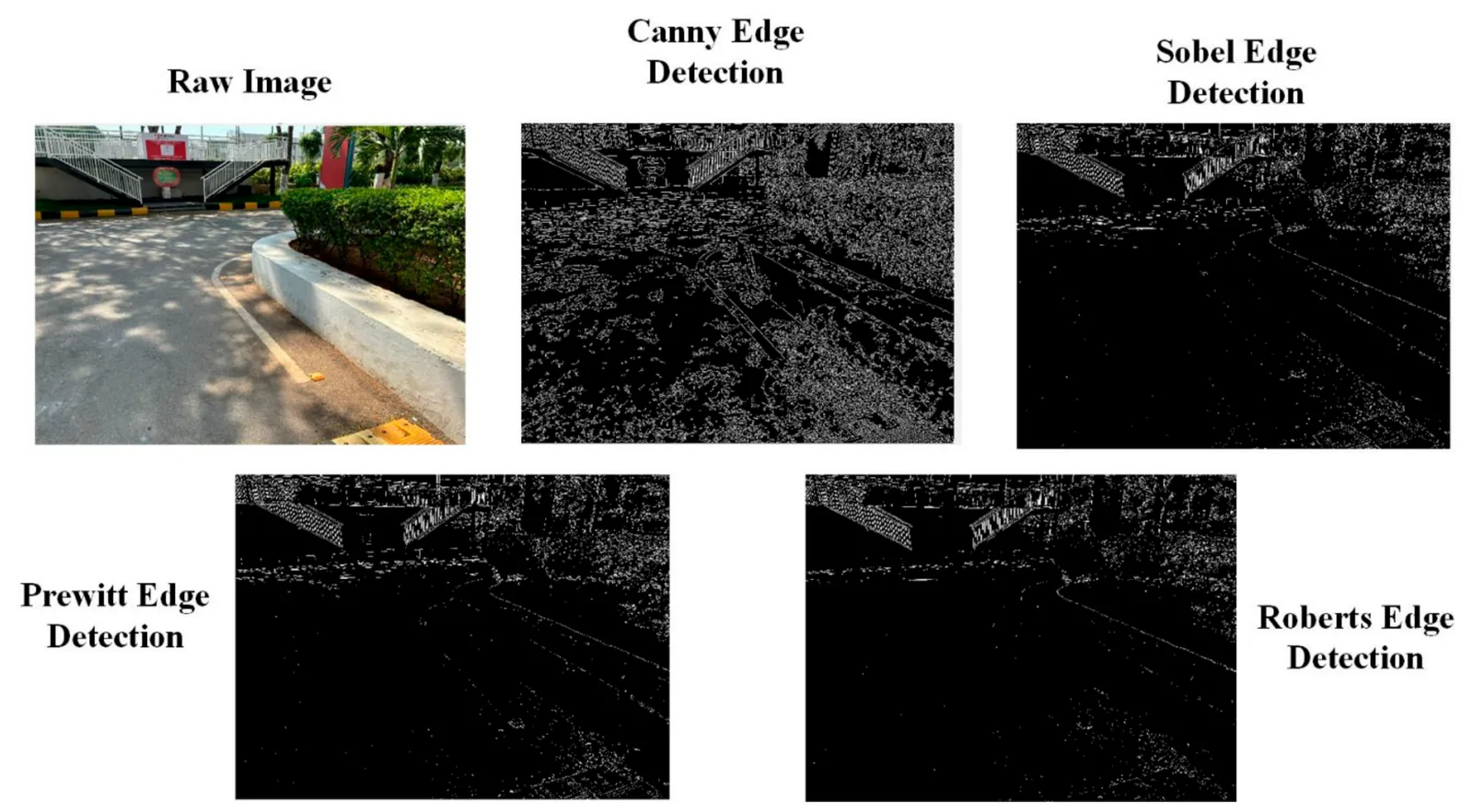
Visual comparison of edge detection techniques.

Four edge detection methods (Canny, Sobel, Prewitt, and Roberts) were applied to the same input images. The resulting edge maps are compared in Figure 5.

The Sobel and Prewitt results contain relatively thick edges, while the Roberts operator shows greater sensitivity to image noise. The Canny output provides thinner and more clearly defined boundaries in the tested images. The precision and recall values in Table 2 are consistent with the visual comparison. Canny gives the highest values among the four methods, with a precision of 0.99 and a recall of 0.98. These results support its selection for the navigation algorithm.

### 3.2. ArUco Marker Detection and Localization

**Figure 6:**
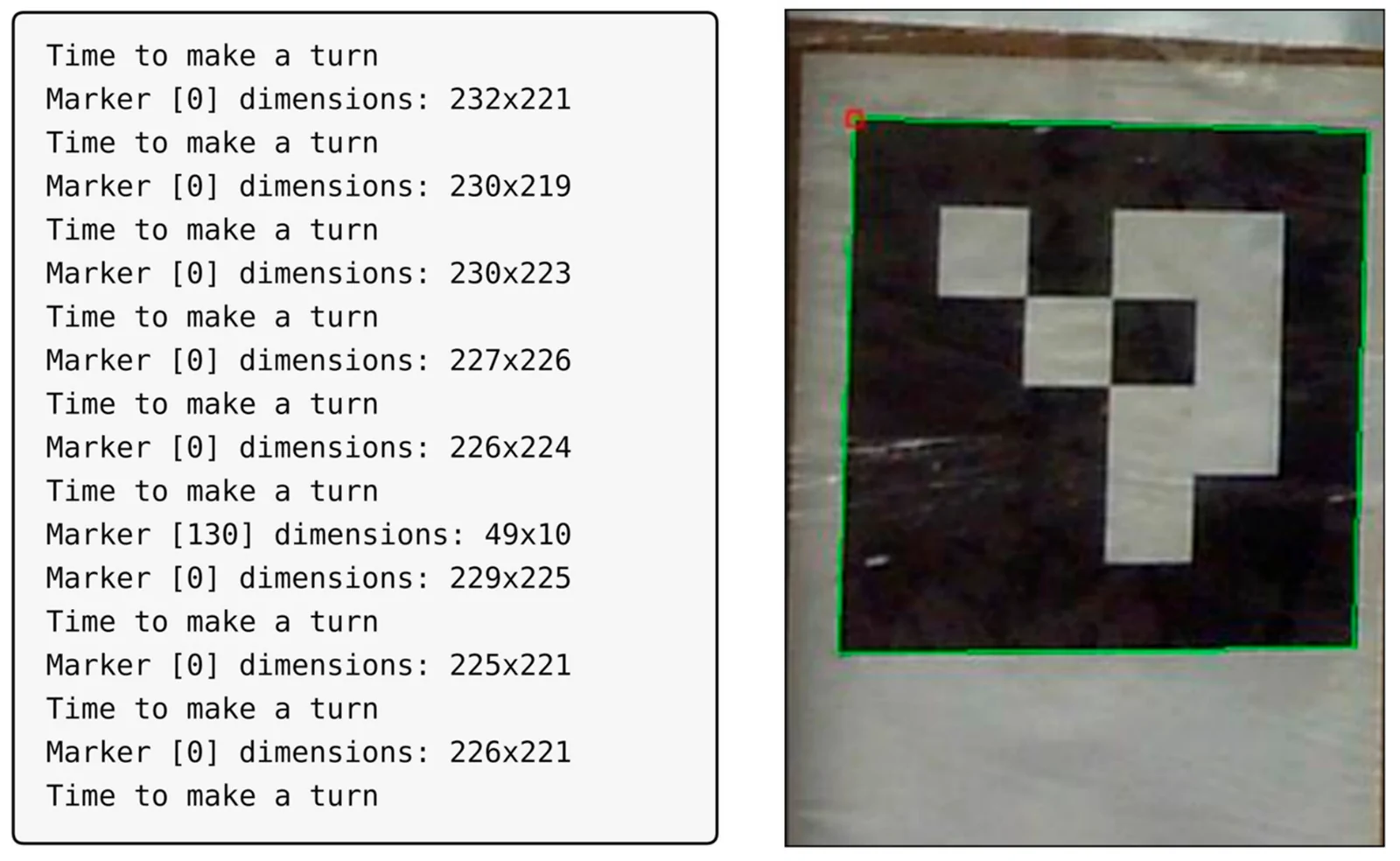
Detection and localization of ArUco marker.

ArUco detection was implemented in Python using OpenCV. The detector provides the marker ID, position, and orientation. Figure 6 shows an example of the detected marker and its centroid calculated using Equation (20). Markers were accepted by the decision stage only when their dimensions were within the specified range. The marker information was then combined with the Canny edge result.

(20) $$\left(x_{c},y_{c}\right)=\frac{1}{4}\sum_{i=1}^{4}\left(x_{i},y_{i}\right)$$

### 3.3. Deep Learning-Based Object Detection

**Figure 7:**
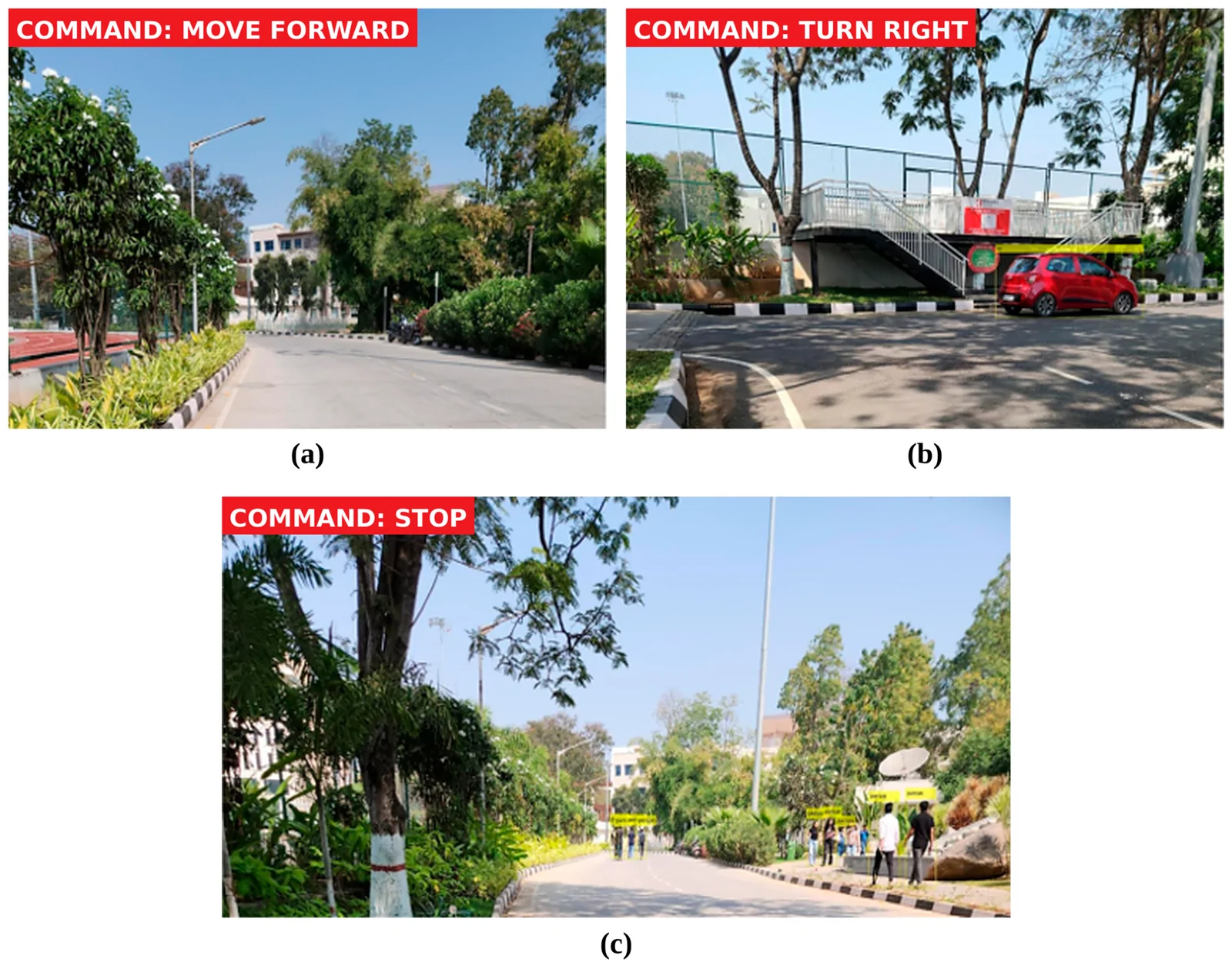
Algorithm response for actionable commands: (**a**) move forward, (**b**) turn right, and (**c**) stop command.

YOLOv4 was evaluated using images containing low-light conditions, occlusion, and background clutter. The model details are given in Section 2; this section reports the observed detection results. A total of 80 field images were collected from EV charging areas, pedestrian crossings, and roundabouts. These images were processed using the pretrained YOLOv4 model (CSPDarknet53-COCO). More than 80% of the recorded detections were pedestrians, followed by motorbikes at approximately 10% and cars at approximately 5%. The remaining detections belonged to other classes identified by the model. The detected objects were passed to the control logic. Depending on the marker, edge, and obstacle conditions, the system generated the commands “STOP”, “TURN RIGHT”, or “MOVE FORWARD”, as shown in Figure 7. The 80 images were used to examine representative object-detection behavior and were not used to claim statistical generalization. YOLOv4 was retained because it was the pretrained detector used in the implemented system. Newer lightweight detectors such as YOLOv8n or YOLO11n may reduce inference time and are suitable candidates for a future comparative study.

The trajectory-control procedure can be extended to traffic-sign recognition by adding a trained sign classifier or detector. Public datasets such as GTSRB and BelgiumTS could be used for training or evaluation. This extension was not implemented in the present study and is therefore identified as future work.

### 3.4. Condition-Based Decision-Making at the Edge

**Figure 8:**
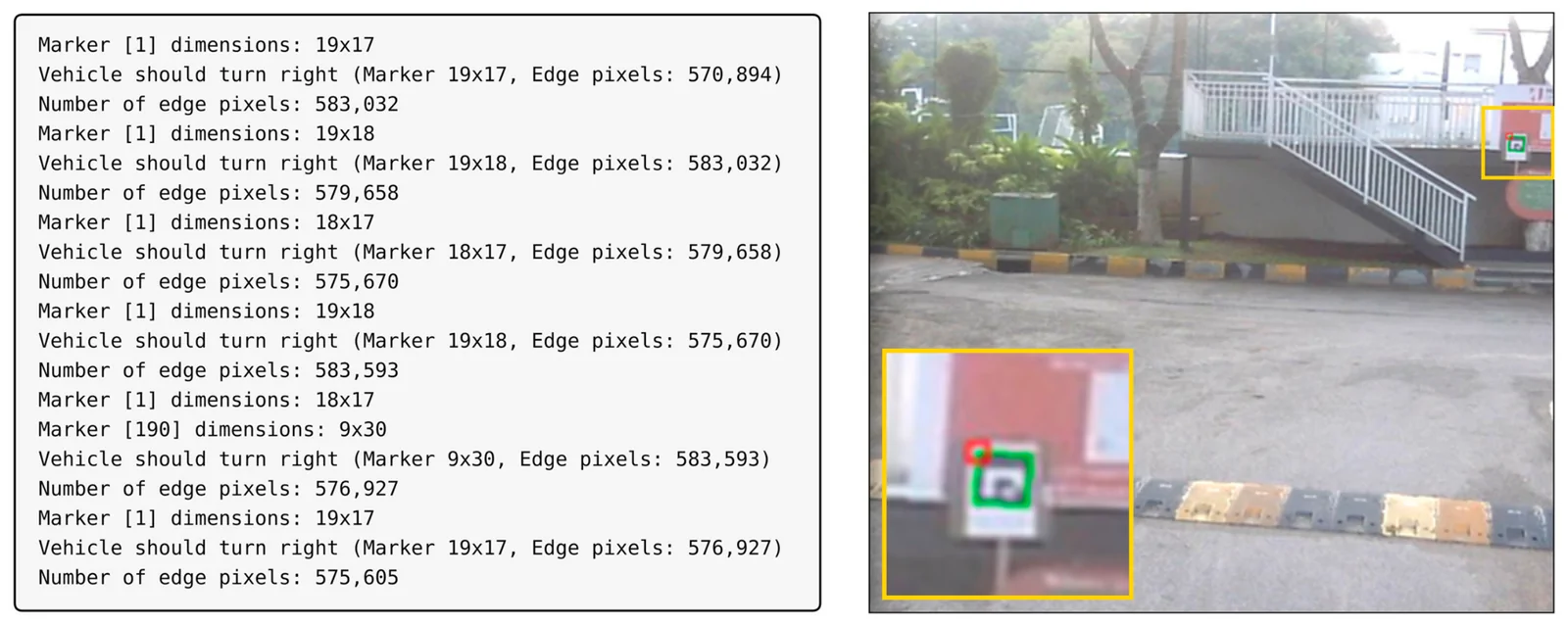
Condition-based decision: vehicle turns right.

The decision loop combines marker dimensions, edge-pixel count, and YOLOv4 obstacle detections before generating a vehicle command. The marker thresholds were set to a minimum width of 17 pixels and a minimum height of 19 pixels. When a valid marker was detected and the edge-pixel count exceeded the selected threshold, the command was “TURN RIGHT”. If the edge count was below the threshold, the vehicle was commanded to move forward. An obstacle detected by YOLOv4 caused a “STOP” command. Figure 8 shows an example of the right-turn decision. In the tested configuration, marker information, edge density, and object detection were processed within the same decision cycle. This allowed the vehicle to change its command when an obstacle was detected.

### 3.5. Standalone Implementation

The hardware implementation was used to verify that the perception commands could be converted into vehicle motion. The physical four-wheel electric vehicle was a commercially available market platform and was not designed or fabricated as part of this study. The research prototype comprises the onboard vision-processing, decision logic, processor-to-microcontroller communication, and motor-control integration added to that platform. The processor sends the selected motion command to the microcontroller, which generates PWM signals for motor velocity and direction. The relation between PWM duty cycle and the motor quantities is given by Equations (21) and (22):

(21) $$PWM=\frac{Duty\;Cycle}{255}\times V_{motor}$$

(22) $$v_{motor}=\frac{PWM}{255}\times v_{max}$$

Changing the PWM duty cycle changed the motor velocity and allowed the vehicle velocity to be adjusted during testing. Communication between the processor, microcontroller, and motor drivers remained stable during the hardware tests. I2C was used for communication between the processor and microcontroller. The processor operates as the master and sends encoded commands such as CMD_TURN_RIGHT = 1. The microcontroller acts as the slave, decodes the command, and generates the corresponding motor-control signals. During the tests, the commands were transferred correctly to the motor-control stage. However, the original campaign did not retain timestamped capture-to-command measurements, stage-wise inference timings, frame-rate logs, or the Jetson power-mode setting. Consequently, a defensible numerical end-to-end latency benchmark cannot be reconstructed from the archived records, and this paper makes no real-time or latency-performance claim.

### 3.6. Deployment Results

**Figure 9:**
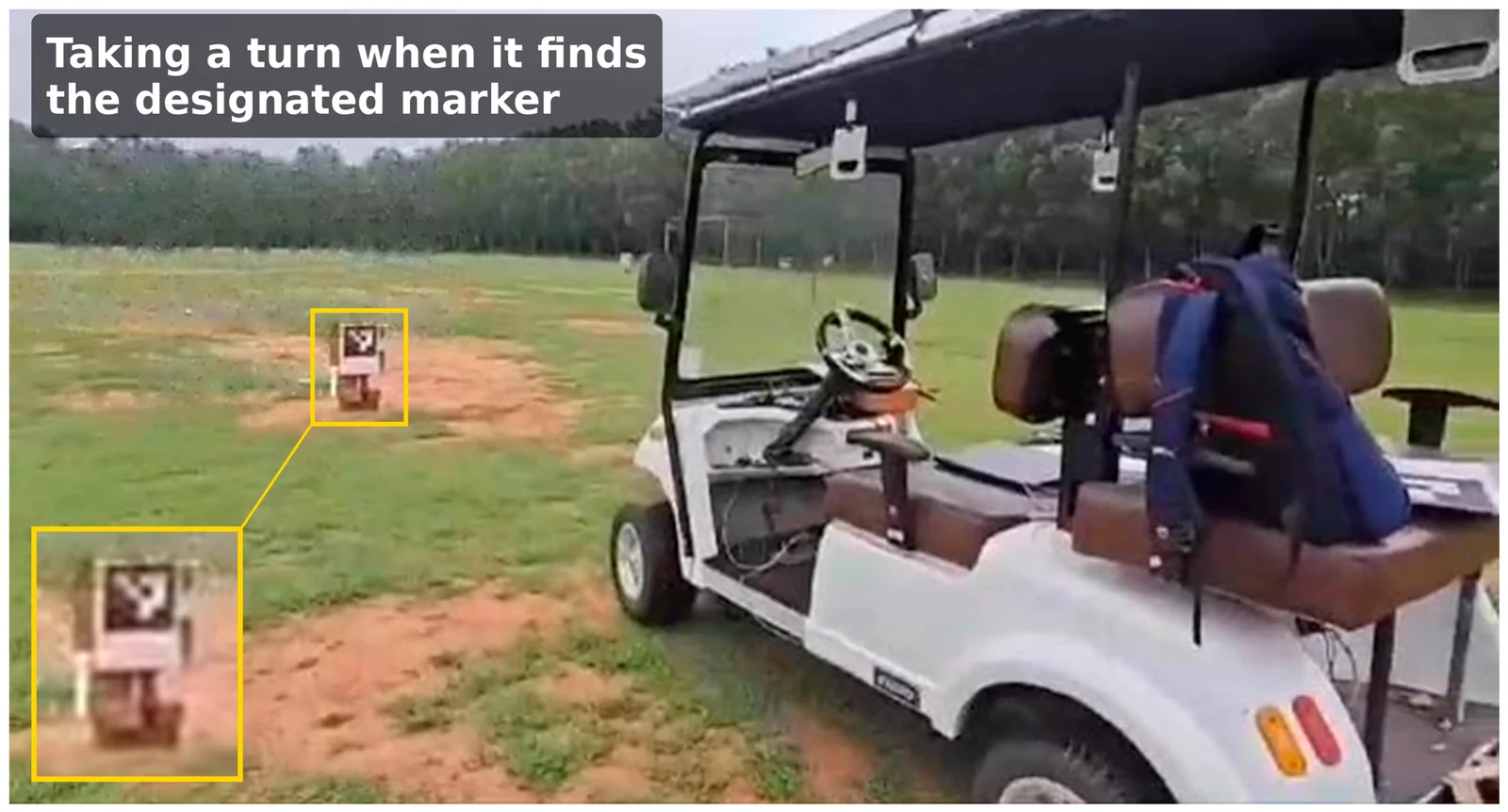
AV navigating the field to scan the marker.

The complete system was first tested in the laboratory and then in a controlled outdoor field, as shown in Figure 9. The processor, microcontroller, motor driver, and vision modules operated together during these tests. The vehicle detected the visual markers and generated forward, turn, and stop commands according to the programmed conditions. Remote emergency control was not included in the reported experiments. For safety-critical deployment, a separate remote-stop or manual override should be added before operation in less controlled environments.

The outdoor tests showed that the vehicle could use the camera information to change its motion. Some degradation was observed when illumination changed, the marker orientation varied, or image noise increased. These conditions affected both Canny edge detection and marker detection. Adaptive thresholds and additional sensor information could be considered in future work to improve performance under these conditions. The reported outdoor tests were performed on a controlled grass/field surface and did not include rain, fog, mud, or other adverse weather conditions. Therefore, robustness under reduced visibility and increased rolling resistance has not been established. Tests on a closed, well-maintained paved area and controlled dynamic-obstacle scenarios would be appropriate next steps.

The current platform is intended for low-velocity controlled environments. Application to urban roads would require additional perception and planning functions, including left/right trajectory selection, traffic-sign recognition, pedestrian interaction, dynamic-obstacle prediction, and stronger localization. Expressway or highway deployment would additionally require higher-velocity vehicle dynamics, longer-range sensing, fail-safe control, and extensive validation. The present experiments should not be interpreted as validation for highway or public-road deployment.

## 4. Conclusions

A hybrid vision-based perception system using ArUco markers, Canny edge detection, and YOLOv4 object detection was implemented as a research prototype on a commercially available four-wheel electric vehicle platform. ArUco was used for localization, Canny for visible road-boundary information, and YOLOv4 for object detection. The main contribution is the decision-level use of these three outputs in a resource-constrained vehicle platform, together with hardware integration through an onboard processor, a microcontroller, and an I2C motor-control interface. The experiments demonstrated the transfer of perception decisions to the motor-control stage. The present study is limited to the tested right-turn navigation scenario and controlled outdoor conditions. The archived experimental record does not contain a documented Jetson power mode or timestamped end-to-end latency measurements; therefore, no numerical real-time performance claim is made. Future work will include left-turn and more complex trajectory commands, newer lightweight object detectors, dynamic-obstacle tests, adverse-weather evaluation, and a dedicated timing campaign using documented power modes and stage-wise latency instrumentation.

## Figures and Tables

**Table 1:** Comparison of existing approaches in autonomous vehicle vision and control systems.

Citation	Methodology	Key Contribution	Challenges
[14]	Embedded vision on Raspberry Pi and Arduino using SIFT, SURF, ORB	Demonstrated feasibility of low-cost AV perception systems	Limited processing power and scalability
[3]	Intelligent decision-making system (IDSS) for adaptive routing	Reduced traffic congestion via real-time data processing	Not optimized for autonomous vehicle navigation
[15]	Deep learning-based real-time object detection	Fast, accurate detection with modest computational demand	Sensitive to illumination and occlusion
[17]	Edge detection and Hough transform-based lane identification	Reliable under varying illumination and weather conditions	Struggles in complex or occluded road geometries
[18,19]	Deep neural network (DNN)-based perception	Improved depth estimation, obstacle detection, and behavior modeling	Requires large annotated datasets and high computational resources
[20]	Learning-based control and collision avoidance	Enhanced adaptability and decision-making in dynamic environments	Computationally intensive for real-time embedded applications
[16,21,22]	Fiducial marker-based localization	Low-cost, robust pose estimation and trajectory guidance	Dependence on marker visibility and lighting conditions
[23]	Classical edge detection for lane and boundary tracking	Effective in low-light and noisy environments	Limited semantic understanding of the scene
[24]	Integration of classical and ML-based methods	Improved robustness under adverse weather conditions	Increased complexity in fusion and parameter tuning
[24]	Vision-guided robotic control for balance and stability	Ensured stable trajectory tracking in dynamic motion	Limited scalability to complex AV systems

**Table 2:** Performance metric comparison between edge algorithms.

Algorithm	Precision	Recall
Canny	0.99	0.98
Sobel	0.64	0.18
Prewitt	0.67	0.19
Roberts	0.48	0.10

## Data Availability

The data presented in this study are available on request from the corresponding author.
